# Lesion Depth Optimization in High‐Power Radiofrequency Ablation: Evaluating Single High‐Power and Combined Very High‐Power Applications

**DOI:** 10.1111/jce.70125

**Published:** 2025-10-02

**Authors:** Hidehiro Iwakawa, Masateru Takigawa, Ryosuke Kato, Junji Yamaguchi, Masaki Honda, Ryo Tateishi, Miho Negishi, Iwanari Kawamura, Kentaro Goto, Takuro Nishimura, Kazuya Yamao, Susumu Tao, Sayaka Suzuki, Takehiro Iwanaga, Shinsuke Miyazaki, Hiroyuki Watanabe, Tetsuo Sasano

**Affiliations:** ^1^ Department of Cardiovascular Medicine Institute of Science Tokyo Tokyo Japan; ^2^ Department of Cardiovascular Medicine Akita University Graduate School of Medicine Akita Japan; ^3^ Division of Advanced Arrhythmia Research Institute of Science Tokyo Tokyo Japan; ^4^ Department of Clinical and Diagnostic Laboratory Science Institute of Science Tokyo Tokyo Japan; ^5^ Japan Small Animal Medical Center Saitama Japan; ^6^ Center for Experimental Animals Institute of Science Tokyo Tokyo Japan

**Keywords:** catheter ablation, ex vivo, in vivo, lesion size, QDOT MICRO, very high‐power short‐duration

## Abstract

**Background:**

A very high‐power short‐duration (vHPSD) radiofrequency (RF) ablation creates shallower lesions, which may be insufficient in thick myocardial regions.

**Aims:**

To clarify an effective ablation strategy following the initial vHPSD application and determine the best approach to create sufficient lesion depth in thick myocardial regions using ex vivo and in vivo models.

**Methods:**

Lesion metrics were compared under various settings: 35 W versus 50 W with the same target ablation index (AI) (Step 1); double vHPSD ablations versus vHPSD followed by AI‐guided ablation (Step 2); double applications (DA) with vHPSD followed by AI‐guided ablation with a target AI of 450 versus single application (SA) with a target AI of 550 at 50 W (Step 3).

**Results:**

Lesion depth was comparable between groups with the same AI but different RF powers. Lesions were significantly deeper in the vHPSD ablation followed by a target AI of 450 compared to double vHPSD ablations (vHPSD + vHPSD, 3.4 [3.1–3.6] mm; vHPSD + AI 450 at 35 W, 4.4 [4.1–4.9] mm; vHPSD + AI 450 at 50 W, 4.5 [4.1–4.9] mm, *p* < 0.001). High‐power SA with a target AI of 550 created significantly deeper lesions than vHPSD + AI 450 (DA vs. SA, 4.5 [3.5–5.3] mm vs. 5.0 [4.1–5.9] mm, *p* = 0.01).

**Conclusions:**

AI‐guided RF applications following vHPSD effectively increased lesion depth more than repeated vHPSD. However, a single high‐power application targeting a higher AI resulted in the deepest lesions. This strategy may be particularly beneficial in thick atrial myocardial regions to enhance lesion durability and improve procedural outcomes.

## Graphical abstract

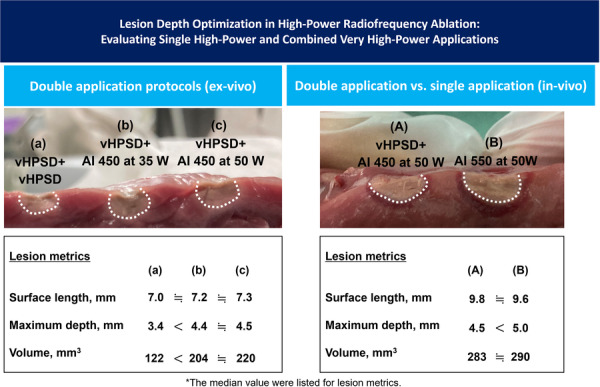

## 1 Introduction

The QDOT‐MICRO™ (Biosense Webster, CA, USA) ablation catheter enables temperature‐controlled, very high‐power and short‐duration (vHPSD) radiofrequency (RF) applications at 90 W for 4 s [1]. While vHPSD significantly reduces procedure and RF times in pulmonary vein isolation (PVI), studies have reported lower first‐pass success rates, potentially due to its limited lesion depth—particularly in thick atrial regions such as the carina [1, 2, 3, 4].

We previously demonstrated in an in vivo experiment that double vHPSD applications delivered with a 4‐ to 8‐second interval created deeper and larger lesions compared to a single vHPSD application [5, 6]. However, it remains unclear to what extent lesion depth can be further increased by delivering additional RF energy after a longer interval, allowing tissue temperature to return to baseline. This scenario may be clinically relevant, as supplementary ablations are often performed at sites of conduction gaps after the initial PV encirclement.

Moreover, it is uncertain whether combining vHPSD with conventional ablation index (AI)‐guided ablation produces deeper lesions than repeating vHPSD applications. Furthermore, the optimal approach to achieve durable transmural lesions in thick atrial tissue is of significant clinical interest.

We aimed to investigate effective RF ablation strategy following vHPSD application that could enhance lesion depth without the effect of thermal latency, and evaluate the optimal approach to create deep lesions in thick tissue using ex vivo and in vivo models.

## 2 Methods

### 2.1 Ablation Catheter and Three‐Dimensional Mapping System

A 3.5 mm tip open‐irrigated contact force (CF)‐sensing ablation catheter (QDOT‐MICRO™) with three microelectrodes and temperature feedback control was used. The nGEN™ RF generator and pump (Biosense Webster, CA, USA) operated a temperature‐flow‐controlled mode with a target temperature of 47°C for 35 and 50 W with an irrigation rate of 4‐15 mL/min, and a temperature‐controlled mode with a target temperature of 60°C for 90 W with a fixed irrigation of 8 mL/min for 90 W [7, 8]. Anatomical locations of the created lesions and catheter stability during RF applications were evaluated using VISITAG^TM^ STABILITY +  module on CARTO 3 system (Biosense Webster, CA, USA), which is effective for RF lesion annotation and for estimating respiration‐compensated catheter motion [9].

Time‐dependent parameter changes of RF power, duration, CF, temperature, AI value, and impedance were automatically collected in a three‐dimensional anatomical mapping system (CARTO 3, Biosense Webster, CA, USA).

### 2.2 Experimental Workflow and Ablation Protocols

**Figure 1:**
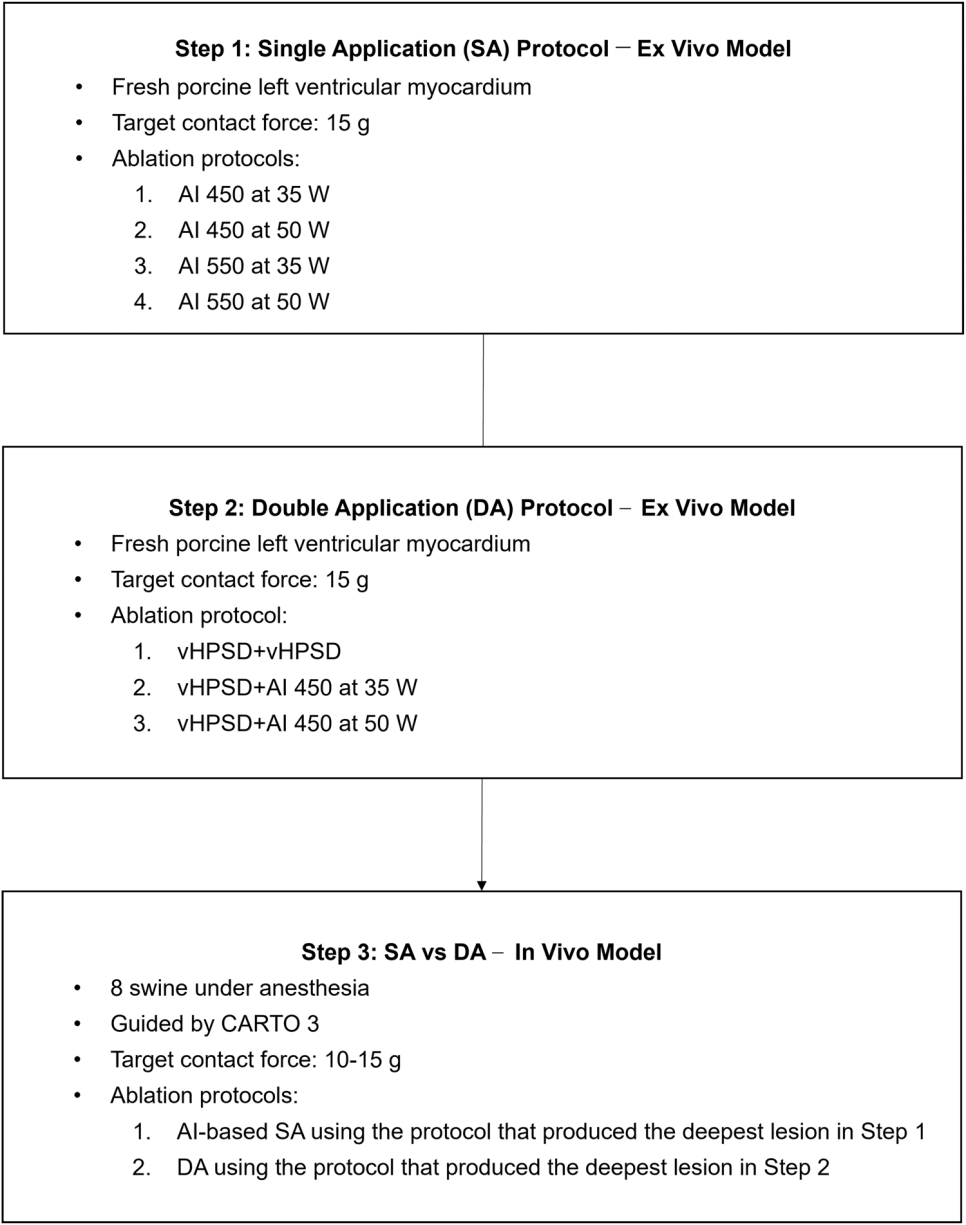
Overview of the experimental workflow.

An overview of the experimental workflow is shown in Figure 1, outlining the step‐by‐step design of this study. The protocol was divided into three main phases: ex vivo comparison of lesions created by single ablations with different AI and power settings (Step 1), evaluation of a double application (DA) strategy using vHPSD in an ex vivo model (Step 2), and in vivo validation of the most effective protocol for achieving deeper lesion in a swine model (Step 3). The target AI values of 450 and 550 were determined based on clinical workflows for ablating thick atrial myocardial regions [10, 11].

### 2.3 Step 1: Ex Vivo Experimental Model and Single Application (SA) Protocol

A section of the porcine left ventricular myocardium excised within 12 h and preserved in a fresh state was placed on a rubber plate and submerged at the bottom of a circulating saline bath containing 5.0 L of saline at 37°C. A flow pump was set to a velocity of 0.2 m/s, replicating atrial flow conditions [12]. Salinity was maintained an impedance level of 100 ± 5 Ω measured by the catheter above the myocardial slab. The ablation catheter was inserted through a plastic tube with a target CF of 15 g, positioned perpendicular to the tissue.

For Step 1, RF energy was delivered using the QDOT‐MICRO™ ablation catheter using the following ablation settings: 1. AI 450 at 35 W, 2. AI 550 at 50 W, 3. AI 550 at 35 W, and 4. AI 550 at 50 W. Steam pop (SP) incidence, ablation parameters, and lesion dimensions were recorded for comparison.

### 2.4 Step 2: Double Application (DA) Protocol Using vHPSD in an Ex Vivo Model

We employed DA strategy, consisting of: 1. double vHPSD ablations (vHPSD + vHPSD), 2. vHPSD ablation followed by ablation with a target AI of 450 at 35 W (vHPSD + AI 450 at 35 W), and vHPSD ablation followed by ablation with a target AI of 450 at 50 W (vHPSD + AI 450 at 50 W). The ex vivo preparations were the same as Step 1. A 1‐min interval, sufficient for tissue temperature to return to baseline, was maintained between the two applications in all DA protocols to simulate the clinical setting of delivering additional RF ablations to address non‐transmural lesions following the initial encirclement around the PVs. Lesion metrics including depth, surface area, and volume were measured and compared.

### 2.5 Step 3: In Vivo Validation in a Swine Experimental Model

The protocol for the in vivo experiment was approved by the Institutional Animal Care and Use Committees of Tokyo Medical and Dental University (A2023‐148C). Eight swine (2 females [25%], aged 2.8–3.5 months; weight 48.3–57.1 kg) were sedated with an intramuscular injection of ketamine hydrochloride (10 mg/kg) and xylazine (2 mg/kg). After sedation, each swine was intubated, following the inhalation of isoflurane, and anesthesia was maintained with 2–3% isoflurane. Ventilation was provided using a respirator (Carestation/Carescape, GE Healthcare, Chicago, IL), with room air supplemented with oxygen. An intravenous sheath was inserted via the internal jugular vein for drug and fluid infusion. Intravenous amiodarone was administered to prevent ventricular arrhythmias. Arterial blood gases were periodically monitored, and ventilator parameters were adjusted to maintain blood gas levels within physiological ranges.

A decapolar catheter was positioned in the coronary sinus (CS) through the internal jugular vein. With the support of a deflectable sheath, right and left ventricular mapping was conducted using the OCTARAY™ Mapping Catheter (Biosense Webster, CA, USA) and CARTO 3 system (Biosense Webster, CA, USA) during CS pacing. RF ablations were then performed using the QDOT‐MICRO™ ablation catheter, targeting a CF of 10–15 g.

Lesion depths created by the SA and DA protocols that produced the deepest lesions in Steps 1 and 2 were compared.

### 2.6 Lesion Size Measurements

Regarding in vivo experimental model, swine were killed and endomyocardial lesions were measured following RF deliveries. The lesion border was defined as the location of a change in tissue color. The myocardium was cross‐sectioned along the surface length at the level of each lesion. Each lesion was measured with a digital caliper with a resolution of 0.1 mm by one observer who was blinded to the lesion protocol. The surface length (*a*), surface width (*b*), maximum length (*c*), maximum depth (*d*), and depth to the maximum length (*e*) of the created lesion were measured. Lesion surface area and lesion volume were calculated from the following formulae: Lesion surface area = *π* * *a*/2 * *b*/2, Lesion volume = (1/6) * *π* * (*c* ^2^*d + e*a ^2^/2) [13, 14, 15].

### 2.7 Statistical Analysis

Continuous variables are expressed as mean ± standard deviation or median and 25th percentile–75th percentile. For categorical variables, data are described as numbers and percentages. To compare two groups, parametric data were analyzed using Student′ t tests and nonparametric data using Mann–Whitney U‐tests. To compare three groups, parametric data were analyzed using one‐way analysis of variance (ANOVA) followed by the post hoc Tukey test, and nonparametric data using the Kruskal–Wallis test followed by the Dunn test. Chi‐square analysis or Fisher′s exact test was used for categorical variables, and the post hoc Holm method was used to compare three groups. Statistical significance was defined as two‐tailed *P*‐values < 0.05. Statistical analyses were performed using StatFlex software version 7.1 (Artech, Osaka, Japan).

## 3 Results

### 3.1 Step 1. Lesion Metrics With AI‐Based SA Protocol

Lesions were created using four protocols: RF power settings of 35 W and 50 W with target AI values of 450 (N = 30 for each) and 550 (*N* = 58 for each). Table 1 summarizes the ablation parameters and lesion characteristics for each setting. The incidence of SP was significantly higher in the AI 550 at 50 W group than in the AI 550 at 35 W group (36.2% vs. 0%, *p* < 0.001), while no SPs were observed in the AI 450 groups at either 35 W or 50 W (*P* = 1.0).

**Table 1:** Ablation parameters and lesion metrics for two target AI settings with RF Power of 35 W and 50 W in the In Vivo model.

	AI 450 at 35 W (*N* = 30)	AI 450 at 50 W (*N* = 30)	*P*‐value	AI 550 at 35 W (*N* = 58)	AI 550 at 50 W (*N* = 58)	*p*
SP, n (%)	0 (0)	0 (0)	1.0	0 (0)	21 (36.2)	<0.001*
Titration, n (%)	22 (73.3)	5 (16.7)	<0.001*	52 (89.7)	29 (50.0)	<0.001*
RF duration, s	21.7 [21.2–22.3]	13.2 [13.1–13.9]	<0.001*	38.6 [36.0–42.0]	22.1 [20.3–25.0]	<0.001*
Baseline impedance, Ω	107 [103–111]	107 [103–111]	0.53	110 [107–113]	109 [106–112]	0.32
Impedance drops, Ω	13.8 [11.5–15.5]	15.6 [13.2–19.1]	0.04*	18.0 [15.9–20.4]	19.7 [18.7–21.4]	0.002*
Maximum temperature, °C	47.6 [45.4–48.1]	44.4 [43.5–47.3]	<0.001*	48.3 [47.8–48.8]	46.8 [45.2–47.6]	<0.001*
Lesion metrics						
Surface length, mm	7.4 [6.9–8.0]	7.2 [6.8–7.7]	0.62	7.0 [5.3–7.8]	7.4 [5.5–8.0]	0.13
Surface width, mm	6.2 [5.8–6.9]	6.1 [6.0–6.4]	0.37	5.7 [5.2–6.6]	6.2 [5.2–6.6]	0.24
Surface area, mm^2^	36.0 [32.5–42.4]	33.4 [32.0–38.2]	0.32	31.8 [21.2–39.1]	34.9 [22.4–41.7]	0.19
Maximum depth, mm	3.8 [3.3–4.1]	3.8 [3.5–4.1]	0.71	5.2 [4.8–5.6]	5.3 [5.0–5.6]	0.42
Volume, mm^3^	172 [142–194]	165 [145–186]	0.85	290 [235–331]	317 [280–354]	0.02*

Abbreviations: AI ablation index, RF radiofrequency

* *p* < 0.05

**Figure 2:**
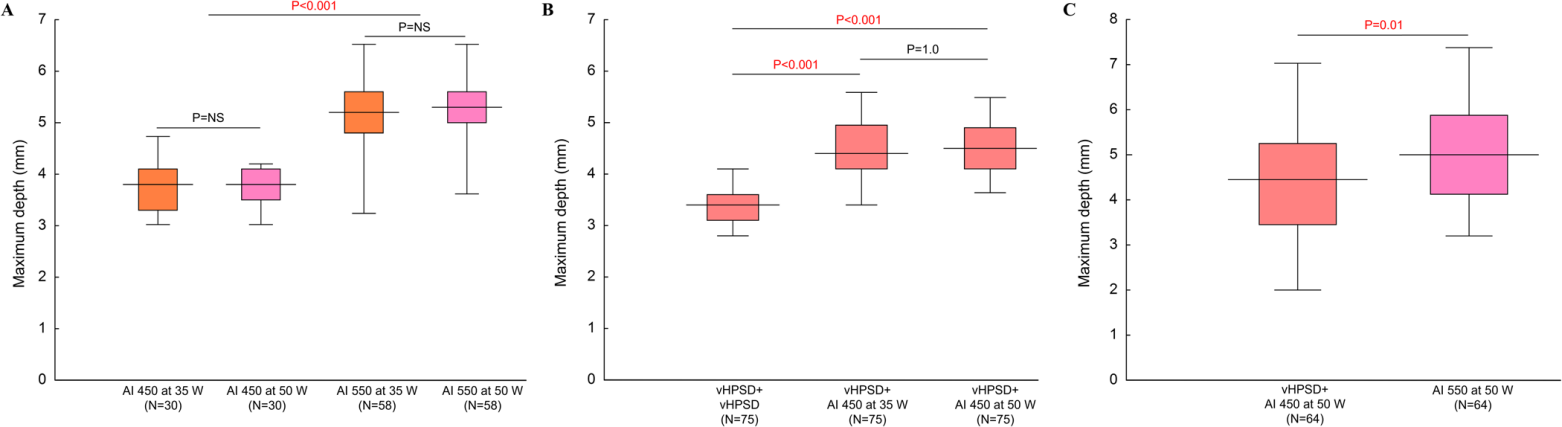
Lesion depths generated by different ablation protocols. (A) Lesion depths generated using the following ablation index (AI) and power settings: AI 450 at 35 W, AI 550 at 50 W, AI 550 at 35 W, and AI 550 at 50 W. (B) Lesion depths resulting from a double‐application strategy, comprising: double very high‐power short‐duration (vHPSD) ablations (vHPSD+vHPSD); a vHPSD ablation followed by an ablation targeting AI 450 at 35 W (vHPSD+AI 450 at 35 W); and a vHPSD ablation followed by an ablation targeting AI 450 at 50 W (vHPSD+AI 450 at 50 W). (C) Lesion depths comparing the vHPSD+AI 450 at 50 W and AI 550 at 50 W protocols.

Lesion metrics, including lesion surface length, width, depth, and area were similar across RF power settings with the same target AI (Table 1). When comparing the AI 450 and 550 groups, lesions were significantly deeper in the AI 550 groups (AI 450 vs. AI 550, 3.8 [3.4–4.1] mm vs. 5.2 [4.9–5.6] mm, *p* < 0.001) (Figure 2A). For AI 450, there was no significant difference in lesion volume between the 35 W and 50 W settings (AI 450 at 35 W vs. AI 450 at 50 W, 172 [142–194] mm^3^ vs. 165 [145–186] mm^3^, *p* = 0.85). For AI 550, lesion volume was larger in the 50 W group than in the 35 W group (AI 550 at 35 W vs. AI 550 at 50 W, 290 [235–331] mm^3^ vs. 317 [280–354] mm^3^, *p* = 0.02) (Table 1).

### 3.2 Step 2. Lesion Characteristics With DA Protocol Utilizing vHPSD

The number of lesions created with vHPSD + vHPSD, vHPSD + AI 450 at 35 W, and vHPSD + AI 450 at 50 W were 75 for each protocol. Table 2 shows the ablation parameters and lesion characteristics for each setting. SPs occurred exclusively in the vHPSD + AI 450 at 50 W group (*p* < 0.001), while the maximum temperature was lowest in this same group (vHPSD + vHPSD vs. vHPSD + AI 450 at 35 W vs. vHPSD + AI 450 at 50 W, 52.4 [49.6–57.9] °C vs. 47.8 [47.1–48.1] °C vs. 44.8 [43.9–47.0] °C, *p* < 0.001).

**Table 2:** Lesion parameters for three double RF application protocols using the vHPSD Ablation setting in the Ex Vivo model.

	vHPSD+vHPSD (*N* = 75)	vHPSD+AI 450 at 35 W (*N* = 75)	vHPSD+AI 450 at 50 W (*N* = 75)	vHPSD+vHPSD vs vHPSD+AI 450 at 35 W *p* value	vHPSD+vHPSD vs vHPSD+AI 450 at 50 W *p* value	vHPSD+AI 450 at 35 W vs vHPSD+AI 450 at 50 W *p* value
SP, n (%)	0 (0)	0 (0)	13 (17.3)	1.0	<0.001*	<0.001*
Titration, n (%)	15 (20.0)	56 (74.7)	17 (22.7)	<0.001*	0.84	<0.001*
RF duration of second application, s	4.0 [4.0–4.0]	22.9 [21.9–24.6]	14.0 [13.0–15.3]	<0.001*	<0.001*	<0.001*
Baseline impedance of the second application, Ω	104 [102–106]	102 [99–105]	104 [103–106]	0.02*	0.97	0.003*
Impedance drops of the second application, Ω	14.0 [13.0–15.0]	11.3 [9.3–12.9]	14.8 [13.3–16.0]	<0.001*	0.66	<0.001*
Maximum temperature, °C	52.4 [49.6–57.9]	47.8 [47.1–48.1]	44.8 [43.9–47.0]	<0.001*	<0.001*	<0.001*
Lesion metrics						
Surface length, mm	7.0 [6.2–7.7]	7.2 [6.0–7.9]	7.3 [6.3–8.0]	0.97	0.54	0.80
Surface width, mm	6.0 [5.7–6.5]	6.0 [5.5–6.6]	6.1 [5.6–6.8]	0.78	1.0	0.67
Surface area, mm^2^	34.3 [28.3–37.8]	33.8 [26.3–39.9]	34.9 [28.0–41.3]	0.99	0.86	0.67
Maximum depth, mm	3.4 [3.1–3.6]	4.4 [4.1–4.9]	4.5 [4.1–4.9]	<0.001*	<0.001*	1.0
Volume, mm^3^	122 [95–149]	204 [169–235]	220 [191–259]	<0.001*	<0.001*	0.18

Abbreviations: AI, ablation index; RF, radiofrequency; vHPSD very high‐power short‐duration,

* *p* < 0.05.

**Figure 3:**
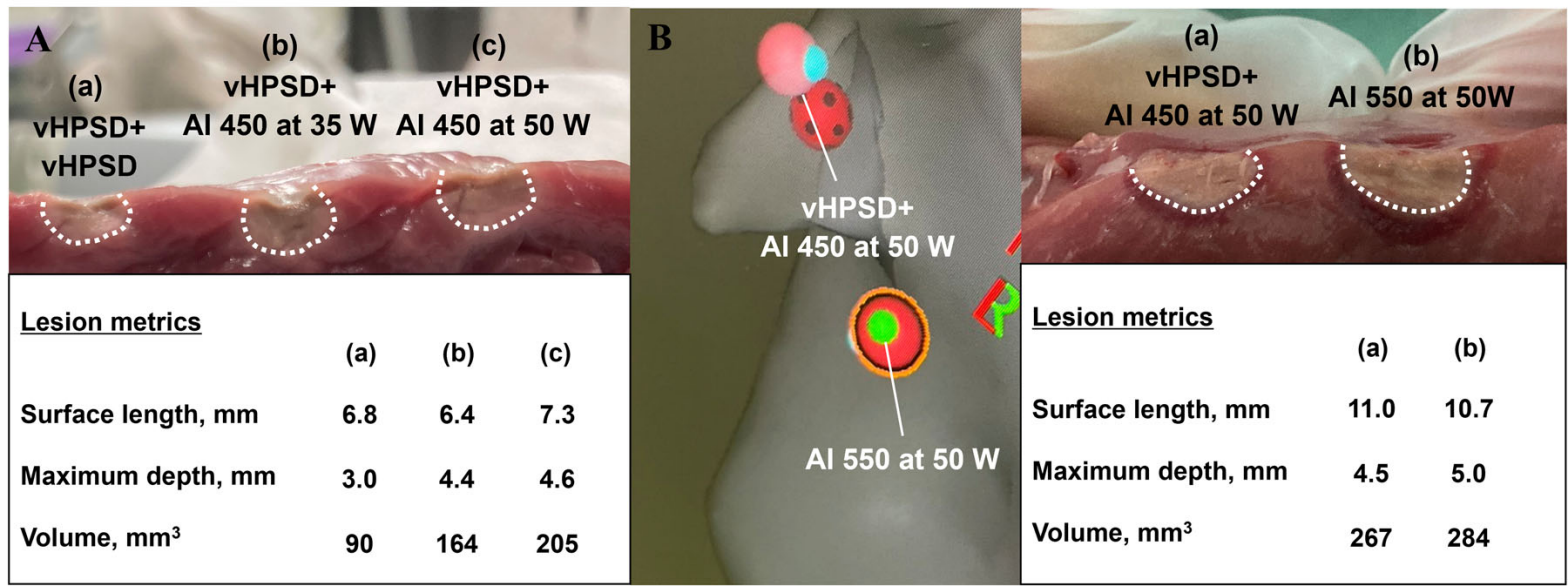
(A) Representative radiofrequency (RF) lesions created using three double application (DA) protocols. Lesion depth and volume are larger in combined very high‐power short‐duration (vHPSD) ablation and ablation index (AI)‐guided ablation protocols compared to double vHPSD ablations, while surface length is similar across three protocols. (B) Representative CARTO image (*left*) and RF lesions comparing combined vHPSD and AI‐guided ablation (vHPSD+AI 450 at 50 W) with single application (SA) at a target AI of 550 at 50 W (*right*). Lesion is deeper with SA than with DA using the vHPSD+AI 450 at 50 W protocol, although surface length and volume are not significantly different.

Lesion surface length, width, and area were similar among the three groups (Table 2). However, lesion depth was significantly shallower in the vHPSD + vHPSD group compared to the other two groups (vHPSD + vHPSD, 3.4 [3.1–3.6] mm; vHPSD + AI 450 at 35 W, 4.4 [4.1–4.9] mm; vHPSD + AI 450 at 50 W, 4.5 [4.1–4.9] mm; vHPSD + vHPSD vs. vHPSD + AI 450 at 35 W, *p* < 0.001; vHPSD + vHPSD vs. vHPSD + AI 450 at 50 W, *p* < 0.001), while lesion depth did not differ between vHPSD + AI 450 at 35 W and vHPSD + AI 450 at 50 W protocols (P = 1.0) (Figure 2B). Similarly, volumes were significantly smaller in the vHPSD + vHPSD group compared to the combined vHPSD + AI 450 groups (vHPSD + vHPSD, 122 [95–149] mm^3^; vHPSD + AI 450 at 35 W, 204 [169–235] mm^3^; vHPSD + AI 450 at 50 W, 220 [191–259] mm^3^; vHPSD + vHPSD vs. vHPSD + AI 450 at 35 W, *p* < 0.001; vHPSD + vHPSD vs. vHPSD + AI 450 at 50 W, *p* < 0.001). Lesion volume did not differ between vHPSD + AI 450 at 35 W and vHPSD + AI 450 at 50 W (*p* = 0.18). Figure 3A demonstrates the representative lesions created by three DA protocols.

### 3.3 Step 3. In Vivo Lesion Assessment With DA and SA Protocols

Based on the Step 1 and 2 findings, Step 3 compared DA (vHPSD + AI 450 at 50 W) and SA (AI 550 at 50 W) protocols in an in vivo swine beating heart model.

After excluding 12 lesions (6 due to mechanical trouble or catheter dislocation, 5 due to difficulty identifying the lesions corresponding to the anatomical mapping system, and 1 due to interruption of RF application caused by ventricular fibrillation), a total of 128 lesions (*N* = 64 for each) including 63 right ventricular (RV) and 65 left ventricular (LV) lesions were created in 8 swine (Table S1). Table 3 depicts the ablation parameters and lesion characteristics in both settings. No SPs occurred in either protocol. RF durations and impedance drops were significantly greater in the SA group than in the DA group (DA vs. SA; RF durations, 18.8 [15.4–24.6] s vs. 28.8 [23.3–44.3] s, *p* < 0.001; impedance drops, 11.0 [9.5–12.6] Ω vs. 13.6 [10.8–15.7] Ω, *p* < 0.001).

**Table 3:** Lesion parameters between combined RF applications with vHPSD and a target AI of 450 and single RF application with a target AI of 550 in the In Vivo model.

	vHPSD + AI 450 at 50 W (*N* = 64)	AI 550 at 50 W (*N* = 64)	*p* value
SP, n (%)	0 (0)	0 (0)	1.0
Titration, *n* (%)	51 (79.7)	48 (75.0)	0.53
Median contact force at 50 W, g	11.5 [9.5–13.9]	11.6 [10.0–15.1]	0.30
RF durations at 50 W, s	18.8 [15.4–24.6]	28.8 [23.3–44.3]	<0.001*
Baseline impedance of the 50 W application, Ω	119 [112–123]	123 [119–129]	<0.001*
Impedance drops at 50 W application, Ω	11.0 [9.5–12.6]	13.6 [10.8–15.7]	<0.001*
Lesion metrics			
Surface length, mm	9.8 [9.0–10.7]	9.6 [8.3–10.7]	0.60
Surface width, mm	6.5 [5.7–7.3]	6.7 [5.9–7.6]	0.43
Surface area, mm^2^	49.3 [42.9–58.7]	50.9 [41.3–59.8]	0.76
Maximum depth, mm	4.5 [3.5–5.3]	5.0 [4.1–5.9]	0.01*
Volume, mm^3^	283 [201–351]	290 [225–425]	0.15

Abbreviations as in Table 2

* *p* < 0.05.

As shown in Figure 2C, although lesion length, width, and area did not differ between the two ablation protocols, the lesion depth volume was significantly greater in the SA group than in the DA group (4.5 [3.4–5.2] mm vs. 5.0 [4.1–5.9] mm, *p* = 0.01). While the lesion volume tended to be larger in the SA group compared to the DA group, the difference was not statistically significant (283 [201–351] mm^3^ vs. 290 [225–425] mm^3^, *p* = 0.15). A representative CARTO image and actual lesions are shown in Figure 3B.

## 4 Discussion

### 4.1 Major Findings

This study evaluated the lesion metrics created by several SA and DA protocols using a temperature‐flow‐controlled ablation catheter in both ex vivo and in vivo models. The key findings are as follows:

1. Lesion characteristics were comparable between the 35 W and 50 W RF power settings when the target AI was the same, although the incidence of SPs and power titration, RF duration, absolute impedance drop, and maximum tip temperature differed between power settings, especially in the ex vivo model.
2. The DA protocol, even with a certain inter‐application interval, produced deeper lesions when combining the vHPSD ablation with AI‐guided ablation, compared to two repeated vHPSD applications.
3. In vivo lesion assessments revealed that SA with a target AI of 550 created significantly deeper lesions than the DA protocol combining vHPSD ablation and AI 450, suggesting that higher target AI‐based SA may be more effective for achieving greater lesion depth.

### 4.2 Deeper Lesion Creation With SA and DA

Our study demonstrated that AI‐guided ablation is more effective in deepening lesions created with the vHPSD ablation than repeating vHPSD ablation twice, even with a significant interval between applications. Additionally, SA with a target AI of 550 created deeper lesions than DA using vHPSD ablation followed by RF applications with a target AI of 450, suggesting that SA with a relatively higher target AI is a practical strategy for creating deeper lesions. Also, lesion size may be more predictable in SA based on the AI‐value, whereas DA using a combined RF strategy can result in more complex lesion formation [16].

Following the introduction of vHPSD technique, some studies have reported comparable first‐pass isolation success rates and durable lesions along PVs using vHPSD compared to conventional 50 W power setting, with shorter procedure and RF times, whereas other researchers have shown that the vHPSD ablation strategy was associated with lower first‐pass PVI rates and higher AF recurrence rates during blanking periods [17, 18]. We recently reported that carina regions were significantly associated with PV gaps after the first encircling around the PV antrum using the vHPSD protocol and the combined approach of vHPSD and AI‐guided ablation improved the first‐pass PVI success rates, suggesting that vHPSD alone may not create transmural lesions, particularly in carina regions [19]. Accordingly, the carina regions were reported to be one of the thickest regions in the LA, with wall thickness ranging from 3.5 to 6.5 mm [20]. These regions are also anatomically covered with multiple muscular layers including the septopulmonary and septoatrial bundles and the ligament of Marshal [21].

We most recently demonstrated that double vHPSD applications with 4–8 s intervals significantly deepened the lesion to approximately 4 mm in an in vivo beating swine heart model [6]. Clinically, additional RF applications to the conduction gaps may be delivered after the completion of the first encircling around the PV [17, 18]. Therefore, we sought to determine a practical method for deepening lesions following initial vHPSD applications with a significant interval. Using a DA protocol with 1‐min intervals, we clearly demonstrated that the second RF applications using fixed AI‐based moderate‐to‐high power strategies significantly deepened the lesions compared with repeated vHPSD applications, resulting in approximately 4.5 mm in depth in an in vivo model. This may be attributed to conductive heating, as RF durations were significantly longer in the vHPSD followed by fixed AI groups than in the double vHPSD group [22].

Nevertheless, lesions created using DA were still shallower than those achieved with SA at a higher target AI of 550, achieving approximately 5 mm in depth. To achieve the predefined target AI values (450 vs. 550), RF application durations were significantly longer in the SA group than in the DA group, leading to greater RF energy transfer to the tissue in the SA protocol. However, given that lesion surface area and volume were similar between the SA and DA groups, the increased RF energy delivery in the SA protocol may not be the sole factor contributing to deeper lesion creation compared with the DA protocol. Another possible explanation is myocardial tissue edema. Several researchers reported that local myocardial edema was observed immediately after thermal ablations [23, 24]. which might weaken the effect of additional applications. Schwartzman et al. reported that atrial wall thickness, assessed with intracardiac echocardiography in an in vivo model, increased significantly by approximately 4 mm immediately after RF applications, and this thickening persisted for up to 1 month [24]. Although this study did not evaluate real‐time changes in wall thickness using intracardiac echocardiography, tissue swelling caused by the initial vHPSD ablation could act as a barrier to further energy transfer and limit lesion deepening during subsequent applications. This tissue swelling could act as a barrier to further energy transfer and deepening the lesions.

### 4.3 Occurrence of SPs Using QDOT‐MICRO™ Ablation Catheter in an Ex Vivo Model

In the ex vivo part, all SPs occurred with the 50 W setting. Accordingly, we recently reported using the QDOT‐MICRO™ ablation catheter in an ex vivo model that RF power of 50 W, as well as low CF, prolonged application time, and perpendicular catheter orientation, was associated with the SP occurrences, while it was significantly infrequent compared with a conventional power‐controlled ablation catheter [25]. By contrast, no SPs were observed during the in vivo part using 50 W setting, suggesting that RF applications at 50 W are safe for short‐to‐moderate durations with the clinical workflow.

This discrepancy may be explained by the irrigation flow rate of the QDOT‐MICRO™ catheter, which is set at 15 mL/min at 50 W. In ex vivo settings, true tissue temperatures may be underestimated due to high irrigation flow, potentially increasing the risk of SPs. In contrast, in vivo conditions may provide greater cooling effects, attributed to catheter instability caused by respiratory and cardiac movements, as well as circulating blood flow perfusing the myocardium. These factors likely prevent excessive tissue heating and reduce the risk of SPs.

### 4.4 Clinical Implications

Our findings provide important histopathological insights for optimizing RF ablation strategies, particularly in challenging anatomical regions such as the carina and areas with higher voltage or longer inter‐lesion distances [17]. While vHPSD ablation is efficient and generally safe, its limited lesion depth may reduce durability, especially in areas with thick atrial myocardium. Our study shows that adding an AI‐guided application on insufficient lesions can efficiently deepen the lesion created with vHPSD. However, a single high‐power application with a higher AI target may offer greater efficiency and more predictable lesion size in thicker myocardial regions.

Starting with an AI‐guided high‐power single application may therefore be a more effective approach, particularly in regions prone to incomplete lesions or conduction gaps. As we previously reported the efficacy of a combined approach using vHPSD and high‐power ablations for PVI [17], this study provides mechanistic and tissue‐level evidence supporting this strategy. This approach may improve first‐pass isolation rates, minimize the need for repeat applications, and lead to better outcomes.

### 4.5 Limitations

This study has several limitations. First, the experiments were conducted using either an ex vivo model or an in vivo model with healthy swine ventricular myocardium. Therefore, the findings may not necessarily generalize to clinical human pathology. However, conducting similar studies in humans with histopathological analysis is challenging. Second, RF applications were performed in thicker tissues including the right and left ventricles because achieving transmural lesions in the thinner atrial myocardium precludes precise measurement of lesion metrics, particularly depth and volume. However, the findings may not be universally applied to atrial tissues. Third, although the locations of the lesions and catheter stability during RF applications were assessed using the VISITAG^TM^ STABILITY+ algorithm, intracardiac echocardiography was not used to identify lesion locations or to evaluate the extent of acute myocardial edema. Fourth, it should be noted that the results of this study are specific to the QDOT‐MICRO™ ablation catheter, which may limit their generalizability to other ablation catheters. Finally, in the in vivo experiments, catheter orientations varied depending on the ventricular regions, which could have influenced the lesion characteristics. Central Illustration 1.

**Central_Illustration 1:**
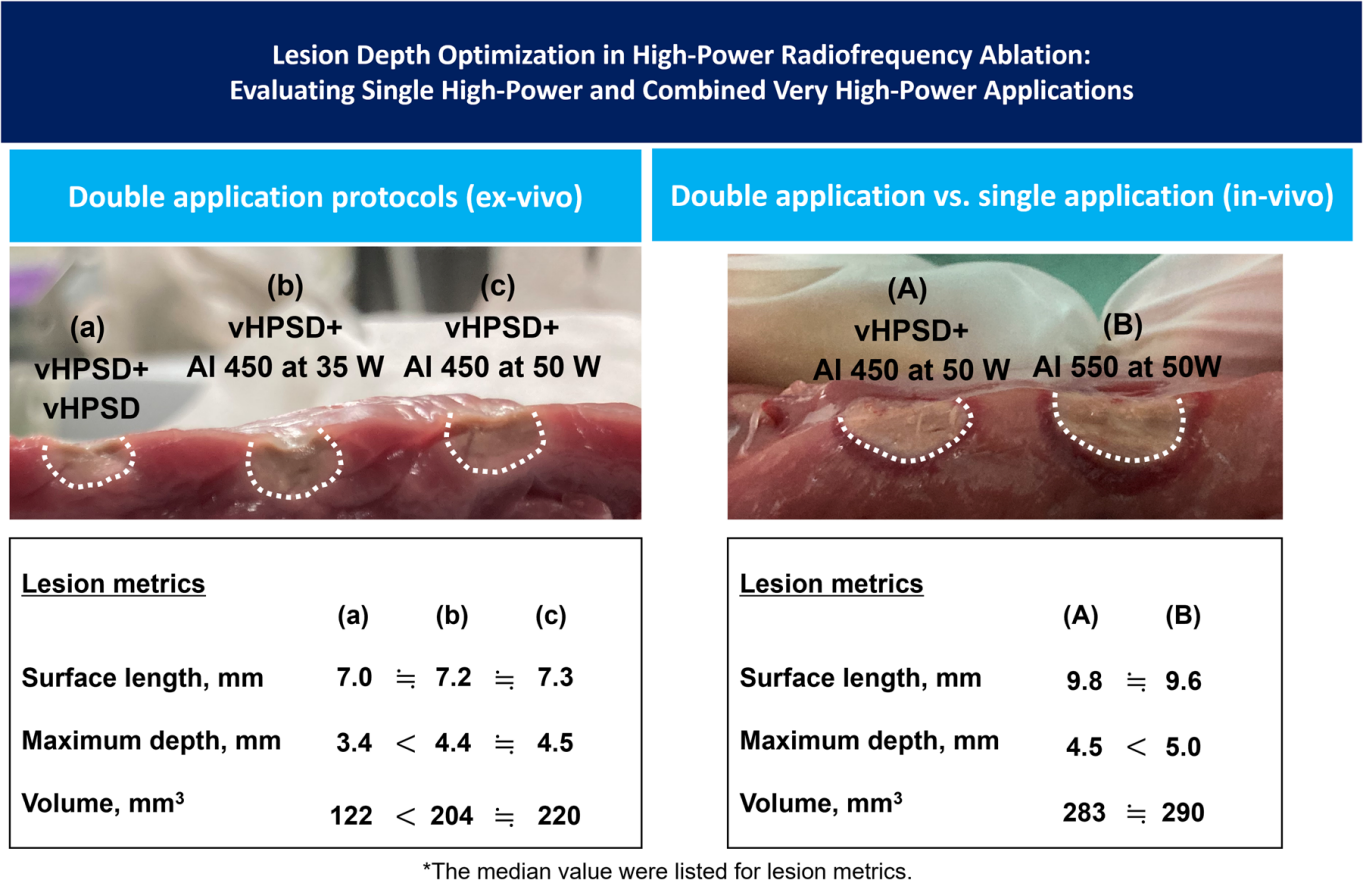
Lesions were significantly deeper with very high‐power short‐duration (vHPSD) ablation followed by a target ablation index (AI) of 450 compared to double vHPSD ablations in an ex vivo model. However, a high‐power application with a target AI of 550 produced significantly deeper lesions than vHPSD+AI 450 in an in vivo model.

## 5 Conclusions

AI‐guided additional application following vHPSD application generated deeper lesions than repeating vHPSD ablations twice, even with a significant interval between applications. An AI‐guided single application strategy can be more effective for generating deeper lesions with higher predictability than a combined approach utilizing vHPSD ablation, particularly in thicker myocardial tissues.

## Ethics Statement

This study was approved by the Institutional Animal Care and Use Committees of Tokyo Medical and Dental University (A2023‐148C).

## Conflicts of Interest

M.T., K.G., and S.M. received endowments from Medtronic Japan, Boston Scientific, Japan Lifeline, and WIN international. The other authors declare no conflicts of interest.

## Supporting information


**Table S1:** Baseline characteristics of swine and lesion counts

## Data Availability

The data that support the findings of this study are available from the corresponding author upon reasonable request. The data underlying this article will be shared on reasonable request to the corresponding author.
