# Supplementary material for: Lesion Depth Optimization in High‐Power Radiofrequency Ablation: Evaluating Single High‐Power and Combined Very High‐Power Applications
Source: J Cardiovasc Electrophysiol. 2025 Oct 2;36(12):3212–21. doi: 10.1111/jce.70125 (PMC12697236; doi:10.1111/jce.70125)
Supplement: Supplementary file 1 — Table S1: Baseline characteristics of swine and lesion counts [file JCE-36-3212-s001.docx]

**Table S1: Baseline characteristics of swine and lesion counts**

|  | Swine No. | Sex | Age, m | Weight, kg | Number of right ventricular lesions  (N=61) | Number of left ventricular lesions  (N=65) |
| --- | --- | --- | --- | --- | --- | --- |
|  | 1 | Male | 3.3 | 52.1 | 4 | 12 |
|  | 2 | Male | 3.0 | 54.1 | 12 | 12 |
|  | 3 | Male | 2.8 | 48.3 | 9 | 8 |
|  | 4 | Male | 3.5 | 52.9 | 3 | 6 |
|  | 5 | Female | 3.5 | 54.1 | 9 | 3 |
|  | 6 | Male | 3.5 | 55.6 | 9 | 8 |
|  | 7 | Female | 3.2 | 57.1 | 7 | 10 |
|  | 8 | Male | 2.9 | 56 | 8 | 6 |
